# Supplementary figures and images for: A Metagenomic Investigation of Spatial and Temporal Changes in Sewage Microbiomes across a University Campus
Source: mSystems. 2022 Sep 19;7(5):e00651-22. doi: 10.1128/msystems.00651-22 (PMC9599454; doi:10.1128/msystems.00651-22)

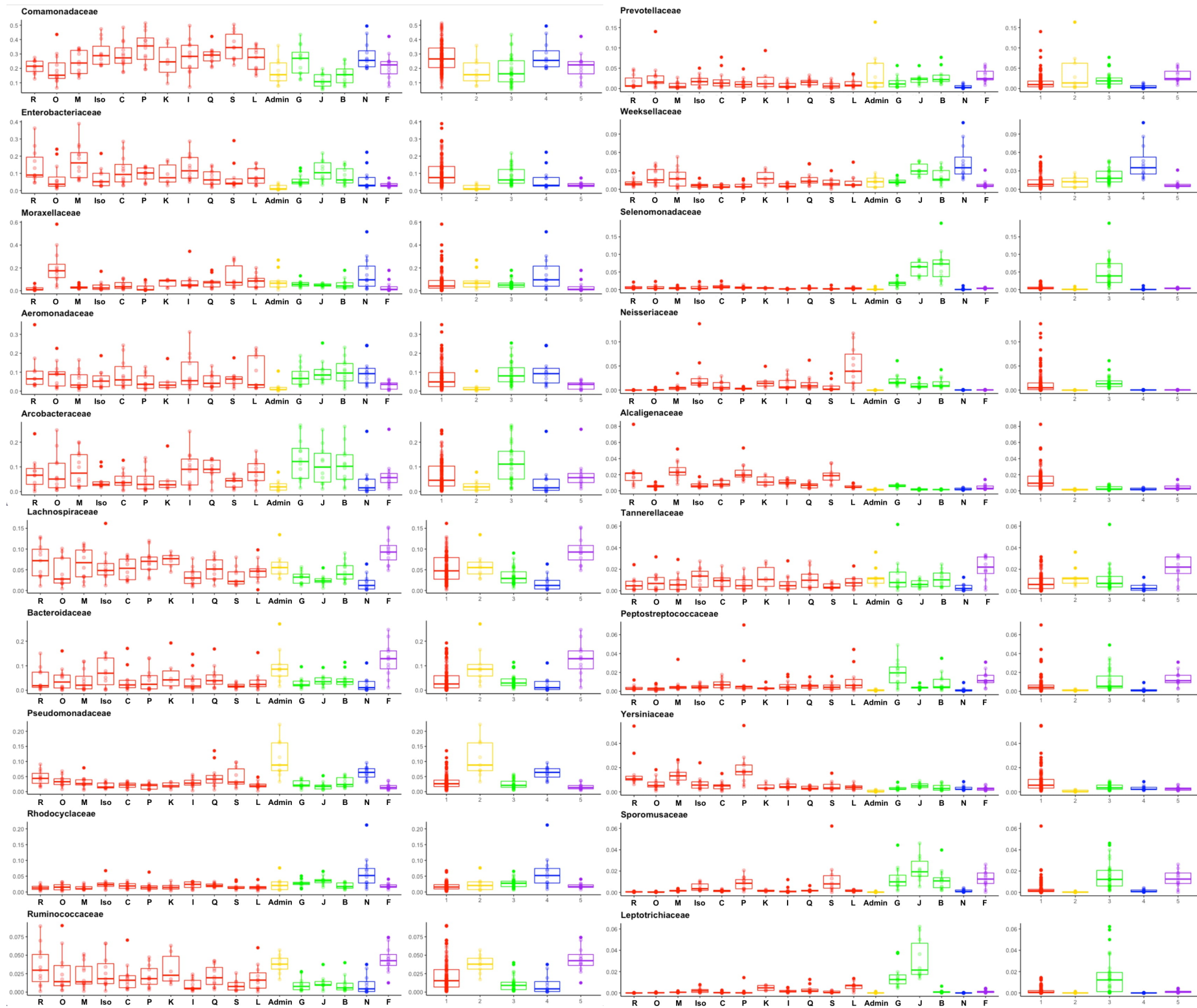

Supplement: FIG S1 [file msystems.00651-22-s0001.pdf]

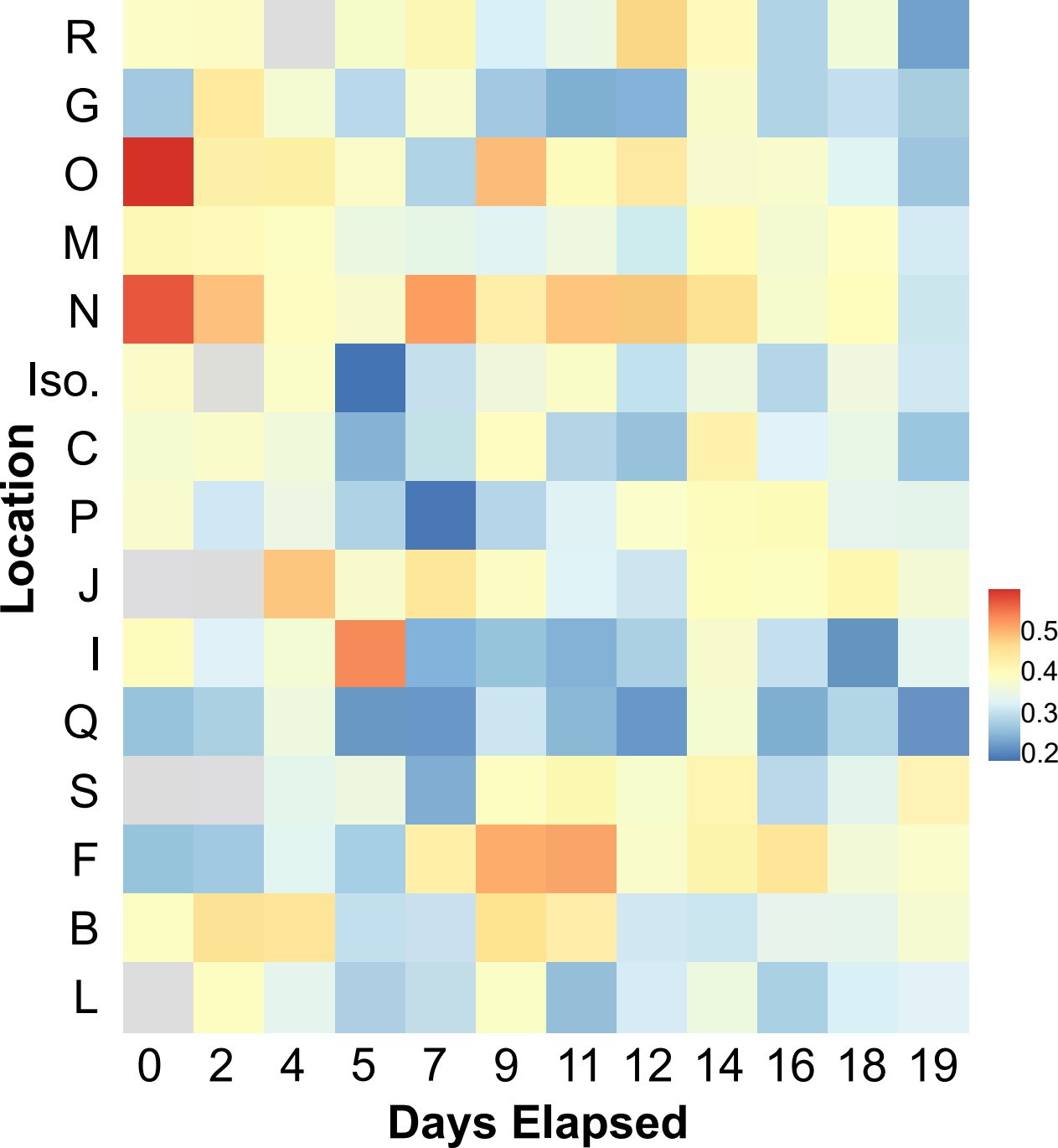

Supplement: FIG S2 [file msystems.00651-22-s0002.pdf]

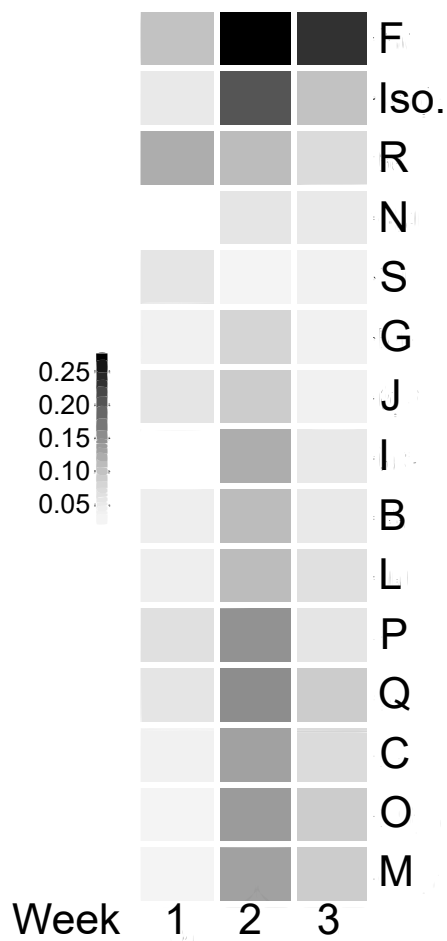

Supplement: FIG S3 [file msystems.00651-22-s0003.pdf]

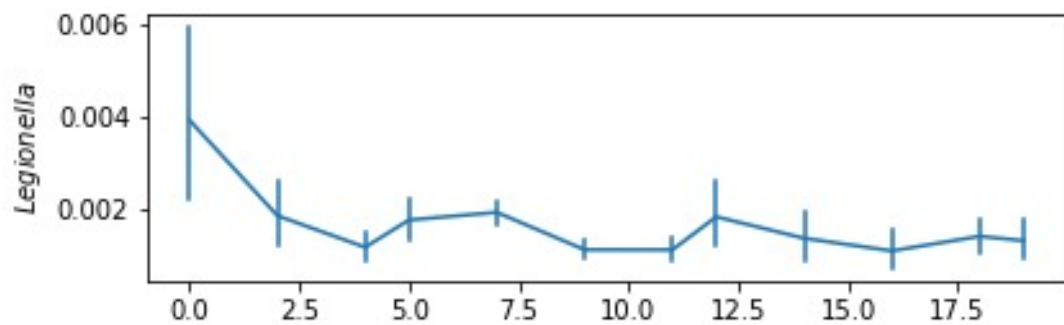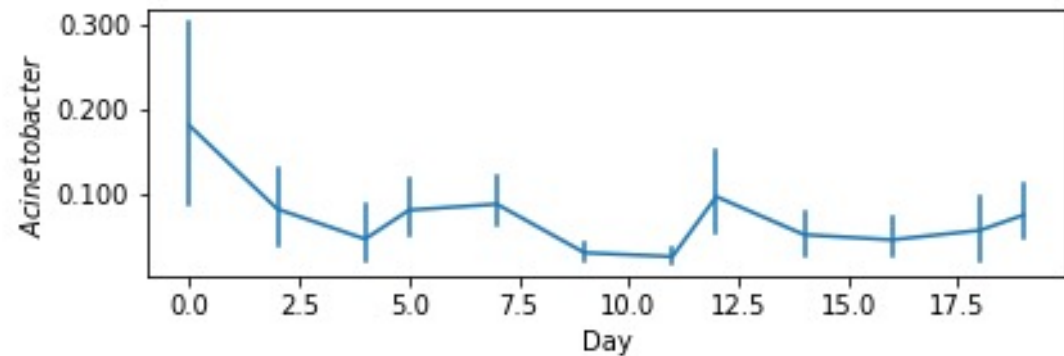

Supplement: FIG S4 [file msystems.00651-22-s0004.pdf]
