# Supplementary material for: A Metagenomic Investigation of Spatial and Temporal Changes in Sewage Microbiomes across a University Campus
Source: mSystems. 2022 Sep 19;7(5):e00651-22. doi: 10.1128/msystems.00651-22 (PMC9599454; doi:10.1128/msystems.00651-22)
Supplement: TABLE S1 [file msystems.00651-22-s0006.pdf]

Supplementary Table 1: Mantel tests and multiple regression on distance matrices (MRM) results for environmental variables associated with temporal patterns in prokaryotic community composition.

Mantel					MRM				
**Site**	**Days R^2**	**Days P**	**TSS R^2**	**TSS P**	**R^2**	**P**	**Var1**	**Var2**	**Var3**
R	-0.048	0.591	-0.048	0.597	0.210	0.084	Days	Precip	pH
G(ABCDEF)	0.282	**0.041**	-0.097	0.760	0.120	**0.010**	Days		
O	0.290	**0.028**	0.215	0.079	0.120	0.150	Days	High Temp	TSS
M	0.368	**0.013**	-0.052	0.629	0.170	**0.020**	Days	Precip	
N	0.380	**0.009**	-0.148	0.862	0.130	**0.021**	Days	High Temp	
Isolation	0.208	0.108	0.487	**0.008**	0.100	0.355	Days	High Temp	Precip
C	0.336	**0.013**	0.331	**0.014**	0.250	0.009	Days	TSS	
P	0.137	0.167	0.449	**0.013**	0.340	**0.004**	pH	TSS	
J	0.454	**0.009**	-0.055	0.612	0.290	**0.005**	Days	High Temp	
I(H)	0.193	0.098	0.363	**0.045**	0.150	0.099	Days	TSS	
Q	0.304	**0.027**	0.301	0.101	0.230	**0.044**	Days	pH	TSS
S	0.178	0.127	0.041	0.442	0.090	0.152	Days	pH	
F	0.457	**0.003**	0.510	0.093	0.510	**0.002**	Days	TSS	
B(A)	0.155	0.116	0.278	**0.043**	0.150	0.147	Days	High Temp	TSS
L(Admin)	0.432	**0.002**	0.447	**0.026**	0.740	**0.032**	Days	High Temp	pH
