# Supplementary material for: A Metagenomic Investigation of Spatial and Temporal Changes in Sewage Microbiomes across a University Campus
Source: mSystems. 2022 Sep 19;7(5):e00651-22. doi: 10.1128/msystems.00651-22 (PMC9599454; doi:10.1128/msystems.00651-22)
Supplement: FIG S5 [file msystems.00651-22-s0005.pdf]

(A)

	L	B	F	S	Ad.	Q	I	J	K	P	C	Iso	N	M	O	G	K
Acinetobacter phage Acj61	0	0.2	0	0	0	0	0	1	14.8	0	0	0	0	0	0	0	0
Aeromonas virus phiO18P	3.6	2.7	0.9	3.9	0.2	2.1	2.3	3.3	0.5	1.6	1.7	1.9	6.5	1	2.3	3.7	1.8
Citrobacter virus Moon	1.5	0	0	4.1	0	0.3	1.2	0	0	0.3	0	1.9	0	0	1.2	0	0
Enterobacteria phage mEp237	0.5	1.4	0.1	0.9	0	0.5	1.5	1.5	0.4	0.9	0.9	0.6	0.3	1.1	0.7	0.8	0.3
Enterobacteria phage phi80	0.8	2.2	0.3	0.5	0.3	0.7	1	2.1	0.3	0.8	0.6	0.7	0.6	1.2	0.9	0.8	1.5
Enterobacteria phage mEp390	0.7	1.2	0	0.3	0	0.4	0.7	1.9	0.2	0.6	0.4	0.4	1.4	0.7	0.6	0.2	0.7
Escherichia phage HK639	1.6	2.5	0.4	1	0.4	1	2.3	3.5	0.8	1.3	1.3	1.3	2	1.9	1.2	1.3	2.4
Escherichia virus P1	0	0	0	1.2	0	0	2.7	0	0	0	6.7	0	0	2.5	1.6	0	0
Salmonella phage Fels-1	0.5	0	0.1	0.4	0	0.6	1.2	0.1	0.8	0.5	2.1	0.6	0.1	1.5	0.2	0.5	0
Salmonella phage Fels-2	1.1	1	0	0.7	0	1	1.4	2.5	0.8	1.2	1.5	1.4	1.6	1.3	1.4	0.7	1.8
Salmonella phage RE-2010	0.8	1.4	0	0.6	0	0.6	1	2.4	0.6	0.9	0.9	0.8	1.1	1	0.9	0.5	1.4
Salmonella phage SEN34	0.7	0.9	0.1	0.3	0	0.4	0.9	1.7	0.2	0.8	0.4	0.6	0.8	0.9	0.5	0.3	1.5
Salmonella phage SEN4 & 5	0.4	2.4	0.1	0.6	0	0.8	1.8	1.3	0.4	2	0.5	1.7	1.9	2	1.2	0.7	1.6
Salmonella phage SJ46	0	0	0	0.5	0	0	1.1	0	0	0	3.7	0	0	0.8	3.6	0	0
Salmonella phage SSU5	2.2	0.6	1.8	0.2	0	2.9	2.4	5.6	1.2	2.2	2.9	1.6	6.4	1.9	1.8	1.8	0.5
Shigella phage SfIV	0.7	0	0.1	0.3	1	0.5	0.5	2.4	0	0	0.4	0.5	0	1.9	0.9	0	0.4
Uncultured crAssphage	69.3	67.7	93.3	56.4	95.5	77.5	60.5	49.3	72	72.1	64.1	75.5	61.7	59.3	66	81.7	66.5
Other	15.2	13.3	2.8	27.5	2.7	9.7	15.9	20	6.6	13	11.3	8.9	13.4	19.2	13.7	6.3	18.1

(B)

	L	B	F	S	Ad.	Q	I	J	K	P	C	Iso	N	M	O	G	K
Unnamed	59.4	63.7	78.5	63.4	75.6	65.6	57.7	61.5	67.3	68.1	63.4	70.8	60.2	61.2	70.1	57.6	60.9
Autographiviridae	0.5	0.2	0.7	1.3	0.3	1	2.6	0.5	0.6	0.8	1.3	2	0.5	0.4	0.4	0.7	1
Mimiviridae	0.2	0.2	0.3	0.2	0.1	0.2	0.2	0.1	0.1	0.3	0.3	0.2	0.1	0.2	0.4	0.9	0.1
Myoviridae	7.9	10.9	4.5	10.3	2	6.9	11.3	11.9	5.8	7.5	10.7	6.6	13.8	13	11.3	8.6	11
Podoviridae	9.7	5.7	9.8	11.4	16	11.3	9.4	5.1	15.6	10.6	10.5	6.7	8.8	10.5	7.5	11	15.3
Siphoviridae	22.2	19.3	6.1	13.4	6	15.1	18.7	20.8	10.6	12.7	14	13.7	16.5	14.7	10.3	21.3	11.7
